# Practice-relevant revision of IPSET-thrombosis based on 1019 patients with WHO-defined essential thrombocythemia

**DOI:** 10.1038/bcj.2015.94

**Published:** 2015-11-27

**Authors:** T Barbui, A M Vannucchi, V Buxhofer-Ausch, V De Stefano, S Betti, A Rambaldi, E Rumi, M Ruggeri, F Rodeghiero, M L Randi, I Bertozzi, H Gisslinger, G Finazzi, A Carobbio, J Thiele, F Passamonti, C Falcone, A Tefferi

**Affiliations:** 1 Research Foundation, Papa Giovanni XXIII Hospital, Bergamo, Italy; 2 Division of Hematology, Papa Giovanni XXIII Hospital, Bergamo, Italy; 3 Department of Experimental and Clinical Medicine, University of Florence, Florence, Italy; 4 Department of Internal Medicine I, Division of Hematology and Blood Coagulation, Medical University of Vienna, Vienna, Austria; 5 Institute of Hematology, Catholic University, Rome, Italy; 6 Department of Hematology Oncology, Fondazione Istituto di Ricovero e Cura a Carattere Scientifico (IRCCS) Policlinico San Matteo, Pavia, Italy; 7 Division of Hematology, S. Bortolo Hospital, Vicenza, Italy; 8 Department of Medicine-DIMED, University of Padua, Padua, Italy; 9 Institute for Pathology, University of Cologne, Cologne, Germany; 10 University Hospital Ospedale di Circolo and Fondazione Macchi, Division of Hematology, Varese, Italy; 11 Division of Hematology, Mayo Clinic, Rochester, MN, USA

Current risk stratification for thrombosis in essential thrombocythemia (ET) is two-tiered and considers low- and high-risk categories based on the respective absence or presence of either age >60 years or history of thrombosis.^1^ In the recent International Prognostic Score for Thrombosis in ET (IPSET-thrombosis), age and history of thrombosis were confirmed as independent risk factors for future thrombosis and the study also identified independent prothrombotic role for cardiovascular (CV) risk factors and *JAK2*V617F mutation.^2^ This model outperformed the two-tiered conventional risk stratification in predicting future vascular events and was not further affected by the recently discovered *CALR* mutation.^3^ In the current study, we re-analyzed the original IPSET-thrombosis data in 1019 patients with WHO-defined ET in whom *JAK2* mutational status was available, to quantify the individual contributions of *JAK2* mutations and CV risk factors in conventionally assigned low- and high-risk ET.

After approval from their respective institutional review boards, seven centers from Italy, Austria and the United States, belonging to the International Working Group for myeloproliferative neoplasm (MPN) Research and Treatment (IWG-MRT), collectively submitted diagnostic and follow-up information on 1220 patients, locally diagnosed with ‘WHO-defined ET'.^4^ Among these, 1019 patients were selected in whom *JAK2* mutational status was available. Objectively proven major arterial and venous events^2^ were reported as rates per 100 patient-years and the difference among groups was assessed by Mantel Cox log-rank test. The Kaplan–Meier product-limit method was used to estimate thrombosis-free survival curves, and the log-rank test was adopted to compare survival curves.

At diagnosis, conventionally assigned low-risk and high-risk groups were significantly different in terms of the frequency of CV risk factors (*P<*0.001) and *JAK2* mutational status (*P<*0.001).

Median follow-up was 6.8 and 5.0 years in conventionally assigned low- and high-risk patients, respectively. Low-dose aspirin and cytoreduction drugs (Hydroxyurea in around 85%) were prescribed in 58 and 41% in low-risk and in 71 and 81% in high risk, respectively. In low-risk patients cytoreduction was started after a median of 18 months since diagnosis because of occurrence of vascular events, age rising to 60 years or progressive thrombocytosis. The overall annual rate of total thrombosis (108 events) in conventionally assigned low- and high-risk patients was 1.11%-pt/y (confidence interval (CI) 0.81–1.52) and 2.46%-pt/y (CI 1.94–3.11), respectively (*P*=0.001), and the difference was mainly due to a higher frequency of arterial thrombosis in high-risk patients (*P<*0.001).

The influence of *JAK2* mutations and CV risk factors on the rate of thrombosis in conventionally assigned low- and high-risk groups is presented in the table.

(i) Conventionally assigned low-risk group. Amongst 506 patients, 200 (40%) displayed neither *JAK2* mutation nor CV risk factors and their annual rate of thrombosis was 0.44%, as opposed to 1.05% in the presence of CV risk factors (*P*=not significant (NS)), 1.59% in the presence of *JAK2* mutation (*P*=0.001) and 2.57% in the presence of both CV risk factors and *JAK2* mutation (*P<*0.001). There was no significant difference when low-risk patients with both *JAK2* mutation and CV risk factors were compared with those with *JAK2* mutation only (*P*=0.217). Figure 1 a shows the time to major thrombosis among patients with the absence or presence of one or two additional risk factors (that is, *JAK2* mutations and CV risk factors).

(ii) Conventionally assigned high-risk group: the absence or presence of one or both of the aforementioned additional risk factors for thrombosis were documented in 111 (22%), 44 (9%), 222 (43%) and 136 (27%) patients, respectively, with corresponding annual rates of thrombosis at 1.44, 1.64, 2.36 and 4.17% (Table). High-risk patients with both risk factors had a significantly higher risk of thrombosis compared with their counterparts without *JAK2* mutations and CV risk factors (*P*=0.011). Figure 1 b shows the probability of events in the groups.

Additional analysis revealed limited enhancement of thrombosis risk by either *JAK2* mutations or CV risk factors or both in patients whose high-risk status was defined by the presence of thrombosis history, regardless of age (*P*=NS). In contrast, the presence of *JAK2* mutations, with or without CV risk factors, might have affected thrombosis risk in patients where high-risk status was defined by age alone (*P*=0.05).

The current study quantifies the individual and combined risk contribution of CV risk factors and *JAK2* mutation in both conventionally defined low- and high-risk ET. The impressively low risk of thrombosis in low-risk *JAK2*-unmutated patients with (1.05% patients/year) or without (0.44% patients/year) CV risk factors clearly distinguishes them from conventionally assigned ‘low-risk' patients with JAK2 mutations, with or without CV risk factors. It is therefore reasonable to further risk-stratify conventionally assigned ‘low-risk' ET into 'very low risk' and ‘low risk' categories, based on the respective absence or presence of *JAK2* mutations (Figure 1c), with the caveat that 'very low risk' disease without CV risk factors is therapeutically approached differently than ‘very low risk ‘ disease with CV risk factors. Such distinction is practically relevant because aspirin therapy might not be necessary in the former group, whereas its efficacy in the latter group and in those with ‘low risk' disease as suggested in a recent retrospective study^5^ might have been undermined by the lack of 24 h therapeutic coverage from the standard once-daily aspirin, especially in *JAK2*-mutated patients with or without CV risk factors.^6, 7^

In high-risk ET, among the two conventional risk factors, the current study delineates thrombosis history as being significantly more detrimental than advanced age. Furthermore, the effect of *JAK2* mutations and CV risk factors in high-risk disease was more apparent, although not statistically significant, in patients whose high-risk disease status was determined by advanced age, whereas the additional impact of these additional risk factors was not significant in high-risk patients with history of thrombosis. This suggests the possible consideration of older patients without thrombosis history or *JAK2* mutations as ‘intermediate-risk' and reserve the 'high-risk' label to patients with thrombosis or to those who are >60 years but also display *JAK2* mutations. In other words, the revised risk stratification scheme might include four categories: ‘very low risk' (no thrombosis history, age ⩽60 years and *JAK2*-unmutated); ‘low risk' (no thrombosis history, age ⩽60 years and *JAK2*-mutated); intermediate risk' (no thrombosis history, age >60 years and *JAK2*-unmutated) and high risk (thrombosis history or age >60 years with *JAK2* mutation). Figures 1 c and d show the thrombosis-free survival probability of patients according to this revised risk stratification.

Treatment recommendations for each one of the above-mentioned new risk categories should be examined in the context of prospective controlled studies. Until results from controlled studies become available, we would not insist on the use of aspirin in ‘very low risk' disease without CV risk factors while we advise once-daily aspirin in ‘very low risk' disease with CV risk factors. We believe that it is reasonable, but not mandated, to consider twice-daily aspirin in ‘low-risk' *JAK2*-mutated patients, especially in the presence of CV risk factors. Similarly, although we encourage the use of cytoreductive therapy in both ‘intermediate risk' and ‘high risk' disease, we would not insist in its use in ‘intermediate-risk' patients, who could be treated, instead, with twice-daily aspirin.

## Figures and Tables

**Figure 1:**
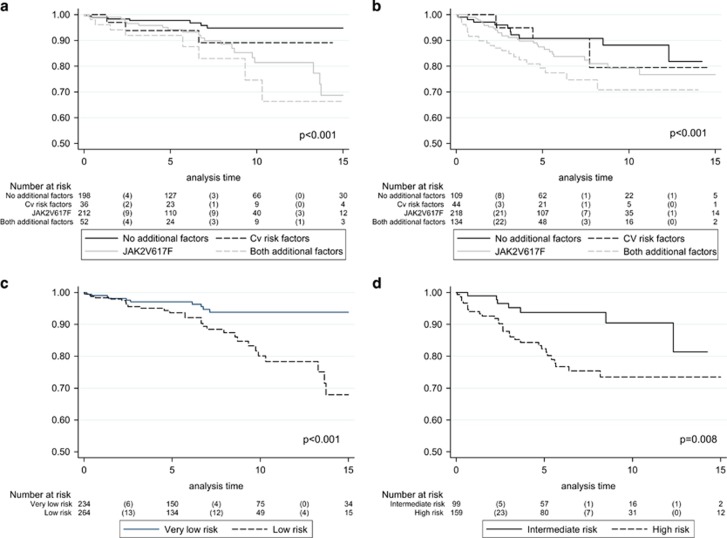
Thrombosis-free survival in conventionally defined low- and high-risk (**a** and **b**) and in the revised risk classification (**c** and **d**). (**a**) Low-risk patients with or without additional risk factors (CV risk factors and JAK2 mutation); (**b**) High-risk patients with or without additional risk factors (CV risk factors and JAK2 mutation); (**c**) ‘Very low risk' (no thrombosis history, age ⩽60 years and JAK2-unmutated); ‘Low risk' (no thrombosis history, age ⩽60 years and JAK2-mutated); (**d**) ‘Intermediate risk' (no thrombosis history, age >60 years and JAK2-unmutated); ‘High risk' (thrombosis history or age >60 years with JAK2 mutation).

**Table 1:** Influence of cardiovascular risk factors and JAK2 mutation on the rate of vascular events in low- and high-risk patients

*Additional risk factors*	N *(%)*	*Event*	*Rate% patients/year (95% CI)*	P-*value*	P-*value*	P-*value trend*
Low risk	506 (50)					
None	200 (40)	7	0.44 (0.21–0.92)	Ref		
Cardiovascular risk factor	36 (7)	3	1.05 (0.34–3.25)	0.220	0.227	
JAK2V617F	213 (43)	21	1.59 (1.04–2.44)	0.001	0.217	
Both	52 (10)	8	2.57 (1.29–5.15)	<0.001	Ref	<0.001

High risk	513 (50)					
None	111 (22)	10	1.44 (0.78–2.68)	Ref		
Cardiovascular risk factor	44 (9)	4	1.64 (0.62–4.37)	0.909	0.067	
JAK2V617F	222 (43)	30	2.36 (1.65–3.38)	0.168	0.082	
Both	136 (27)	25	4.17 (2.82–6.17)	0.011	Ref	0.005
